# Non-alcoholic steatohepatitis incidence in patients with psoriasis vulgaris

**DOI:** 10.1007/s10354-025-01107-6

**Published:** 2025-08-29

**Authors:** Hanaa Mohamed Elhanon, Rabie B. Atallah, Ramadan M. Eldahshan, Saad El Deen Mohamed, Mohamed L. Elsaie

**Affiliations:** 1 Department of Dermatology and Venereology, Itay El-Baroud Hospital, El Beheira, Egypt; 2 Department of Dermatology and Venereology, Damietta Faculty of Medicine, Al-Azhar University, Damietta, Egypt; 3 Department of Internal Medicine, Damietta Faculty of Medicine, Al-Azhar University, Damietta, Egypt; 4 Department of Dermatology, Medical Research and Clinical Studies Institute, National Research Centre, Giza, Egypt

**Keywords:** Abdominal ultrasonography, Liver disease, Metabolic disease, Liver stiffness, Skin

## Abstract

**Background and aims:**

Non-alcoholic steatohepatitis (NASH) is a severe form of non-alcoholic fatty liver disease (NAFLD), characterized by hepatic inflammation and damage due to fat accumulation. Psoriasis patients show higher NASH incidence due to overlapping risk factors like obesity and insulin resistance. The study aimed to determine the incidence of non-alcoholic steatohepatitis in patients with psoriasis vulgaris.

**Methods:**

This cross-sectional observational study included 80 adult patients diagnosed with psoriasis vulgaris. Psoriasis severity was assessed using the Psoriasis Area and Severity Index (PASI) score. All participants underwent abdominal ultrasonography to assess liver steatosis. Those with significant findings suggestive of fatty liver were further evaluated with transient elastography (FibroScan, EchoSens, Paris, France) to determine liver stiffness and controlled attenuation parameter (CAP) values.

**Results:**

Among psoriatic patient diagnosed with NASH, the mean PASI score was 8.8 ± 3.6 which was higher than those diagnosed with NAFLD and non-NASH non-NAFLD patients (6.7 ± 4.5 and 7.1 ± 3.9, respectively); however, this difference was not statistically significant (*P* = 0.19). The percentage of moderate and severe psoriasis was higher in NASH patients (68.75%) compared to NAFLD and non-NASH non-NAFLD subjects (57.1% and 46.5%, respectively).

**Conclusion:**

NASH and NAFLD are linked to psoriasis severity and systemic metabolic dysfunction. Future studies with larger cohorts and prospective designs are needed to validate these findings and explore underlying mechanisms.

**Graphic abstract:**

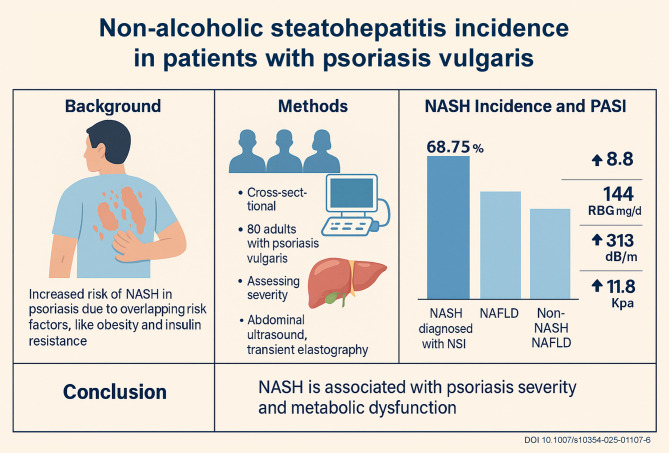

## Introduction

Non-alcoholic steatohepatitis (NASH) is a severe form of non-alcoholic fatty liver disease (NAFLD), characterized by hepatic inflammation and damage due to fat accumulation. It can progress to cirrhosis and hepatocellular carcinoma, posing significant health risks [1].

Psoriasis vulgaris, a chronic inflammatory skin disorder, has been increasingly associated with systemic comorbidities, including metabolic syndrome and cardiovascular diseases. A notable incidence of NASH among patients with psoriasis vulgaris indicated a potential link between the two conditions [2].

The prevalence of NAFLD in the general population is approximately 25%, with NASH affecting a subset of these individuals. In contrast, patients with psoriasis exhibit a higher prevalence of NAFLD, ranging from 44% to 65%, suggesting that psoriasis patients are at an increased risk for developing NAFLD and its more severe form, NASH [2].

Several factors contribute to the heightened incidence of NASH in psoriasis patients. Both conditions share common risk factors, such as obesity, insulin resistance, and dyslipidemia, which are components of metabolic syndrome, thus, highlighting the need for awareness and monitoring of liver health in these patients [2].

A number of proinflammatory cytokines involved in the pathogenesis of psoriasis, e.g., as tumor necrosis factor-alpha (TNF-α) and interleukin-17 (IL-17), have been implicated in the development of hepatic steatosis and inflammation. Moreover, the severity of psoriasis appears to correlate with the risk and progression of NAFLD/NASH. Patients with more extensive skin involvement or higher Psoriasis Area and Severity Index (PASI) scores have been observed to have a greater likelihood of developing NAFLD [3]. Hepatic ultrasonography is widely used for screening of fatty liver disease in asymptomatic patients, carrying a positive predictive value of 62% and a sensitivity of 100% in the detection of steatosis of 33% or greater [3].

Non-invasive imaging techniques, such as transient elastography, can aid in assessing liver fibrosis and steatosis. It allows rapid and noninvasive measurement of steatosis and fibrosis simultaneously, and the median values are used to quantify liver fibrosis and steatosis. The FibroScan (Echosens, Paris, France) machine introduced in 2003 using transient elastography uses two parameters: the controlled attenuation parameter (CAP) to estimate steatosis and the liver stiffness measurement (LSM), measured in kilopascal (kPa), to estimate liver fibrosis [4].

The aim of this study was to determine the incidence of non-alcoholic steatohepatitis in patients with psoriasis vulgaris exploring their shared pathophysiological mechanisms, epidemiological trends, and potential implications for clinical management.

## Patients and methods

This cross-sectional observational study was conducted at Al-Azhar University Hospital (Damietta) over a period from January to November 2024 year, after obtaining approval from the institutional ethics committee. Written informed consent was obtained from each participant. The study adhered to the Declaration of Helsinki and followed all ethical guidelines for human research. The study included 80 adult patients diagnosed with psoriasis vulgaris, aged 18 years and older, who attended the dermatology outpatient department during the study period. We excluded patients with any of the following criteria: 1) History of liver diseases (e.g., viral hepatitis, autoimmune hepatitis, Wilson’s disease, or primary biliary cirrhosis). 2) Patients on systemic medications known to induce liver steatosis (e.g., corticosteroids, methotrexate, tamoxifen). 3) Patients with any reported alcohol consumption were excluded. Only individuals with confirmed complete abstinence were enrolled, based on World Health Organization alcohol use screening criteria. 4) Pregnancy or lactation. In addition, social, occupational, and medication histories were obtained to exclude confounding hepatic factors such as alcohol consumption, hepatotoxic drugs, or industrial exposures. Patients with a prior diagnosis of psoriatic arthritis (PsA) or clinical features suggestive of PsA were excluded to maintain a focus on cutaneous manifestations.

## Sample size calculation

A convenience sample technique was used. All attendants who matched the inclusion criteria were recruited until the desired sample size was achieved. To calculate the sample size, we used the following formula *n* = Z^2^ P (1–P)/d^2^, where *n* is the sample size, Z is the statistic corresponding to level of confidence (95%), P is expected prevalence which was 14.1% in the study of Maybury et al. [5], and d is precision (0.05). Based on the above data the smallest sample size was 44 patients; however, after compensating for a possible 10% dropout rate, the required sample size should be at least 50 patients.

## Data collection

All patients were subjected to complete medical history taking (age, sex, comorbidities, psoriasis duration, severity, and treatment history), clinical examination including (body mass index [BMI] and waist circumference), and laboratory investigations including liver function tests (alanine transaminase [ALT], aspartate transaminase [AST], prothrombin time [PT], albumin, and bilirubin), lipid profile mainly (total cholesterol, triglycerides), random blood glucose (RBG), and complete blood count (CBC). Laboratory values were interpreted using institutional reference ranges. Psoriasis severity was assessed using the Psoriasis Area and Severity Index (PASI) score [6]. All participants underwent abdominal ultrasonography to assess liver steatosis. Those with significant findings suggestive of fatty liver were further evaluated with transient elastography (Fibro Scan®; EchoSens, Paris, France) to determine liver stiffness measurement (LSM) and controlled attenuation parameter (CAP) values. At least 10 valid measurements were obtained in each patient.

A successful examination was defined as 10 validated measurements and an interquartile range (IQR) to median ratio of ≤ 30% was considered reliable and used for the final analysis. CAP values > 248 dB/m were considered indicative of significant hepatic steatosis, and liver stiffness values > 7.9 kPa were used as a threshold suggestive of NASH in the context of metabolic dysfunction and sonographic evidence of fatty liver. These thresholds were based on established FibroScan® cut-offs validated in metabolic liver disease. Patients with findings indicative of advanced fibrosis or cirrhosis were excluded from the study.

## Outcome measure

The primary Outcome was the incidence of NASH in patients with psoriasis vulgaris. Secondary outcomes studied the association between psoriasis severity and NASH as well as identification of clinical and biochemical predictors of NASH.

## Statistical analysis

Statistical analysis was performed with SPSS statistical software (version 26, IBM, Armonk, NY, USA). The normality of the data was tested by the Kolmogorov–Smirnov test. Qualitative data were presented as numbers and percentages and were compared by the Chi square test, or Fisher exact test. Quantitative data were presented as mean and standard deviations and were compared by the independent t test. As a result, the *p*-value will be considered significant at the level of < 0.05.

## Results

**Fig. 1:**
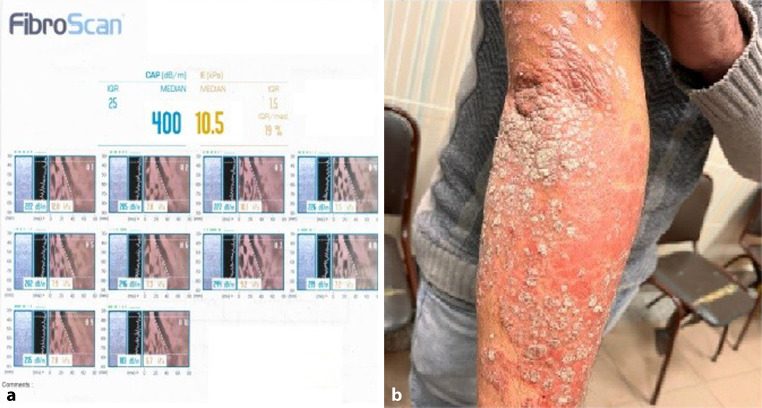
Correlation between **a** transient elastography: (Fibroscan, Echosens) displaying reconstructed views of the wavefront that passes through the liver from the handheld probe and controlled attenuation parameter (CAP) measurements displayed on the left-hand side and **b** case of severe psoriasis

**Fig. 2:**
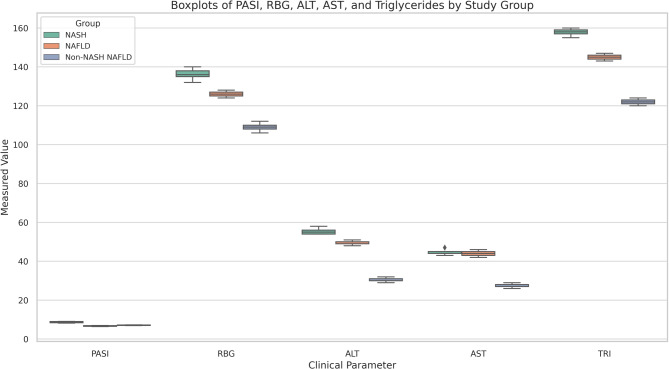
Boxplots of clinical parameters across study groups (NASH, NAFLD, and non-NASH non-NAFLD) illustrating the distribution of Psoriasis Area and Severity Index (PASI), random blood glucose (RBG), alanine aminotransferase (ALT), aspartate aminotransferase (AST), and triglyceride (TRI) levels among patients diagnosed with NASH, NAFLD, and non-NASH non-NAFLD. Higher PASI, RBG, and liver enzyme values are noted in the NASH group, supporting the metabolic and inflammatory link between psoriasis severity and liver dysfunction

A total of 80 participants were included in the current study. The mean age was 42.9 ± 10.6 years with a range of 20–64 years. According to their gender distribution, 47 participants (58.8%) were males, and 33 participants (41.3%) were females. The mean BMI was 28.6 ± 4 kg/m^2^ with a range of 20–36. Laboratory data and profile did not show any significant variance while the mean PASI score of the studied population was 7.8 ± 4 with a range of 1–16. Of the included subjects, 16 patients (20%) were diagnosed with NASH, 21 patients (26.2%) were NAFLD, and 43 patients (53.8%) were non-NASH non-NAFLD. Seven patients had a history of methotrexate use, with a median cumulative dose of 1500 mg. None were on systemic therapy at the time of liver evaluation. Among the study cohort, 26.2% had type 2 diabetes mellitus, 31.2% had hypertension, and 17.5% were on lipid-lowering agents. Use of antidiabetics and statins was documented and included in subgroup comparisons (Tables 1, 2 and 3; Figs. 1 and 2).

**Table 1:** Demographic data of the participants

Variables	Mean ± SD or *N* (%)
Age (years) Mean ± SD	42.9 ± 10.6
Range	20–64
Gender—males	47 (58.8%)
Gender—females	33 (41.3%)
BMI (Kg/m^2^) Mean ± SD	28.6 ± 4
Range	20–36
PASI score Mean ± SD	7.8 ± 4
Range	1–16
Diabetes mellitus—*n* (%)	21 (26.2%)
Hypertension—*n* (%)	25 (31.2%)
On lipid-lowering drugs—*n* (%)	14 (17.5%)
On antidiabetic medications—*n* (%)	18 (22.5%)
Prior methotrexate use—*n* (%)	7 (8.8%)
Cumulative methotrexate dose (mg) median (range)	1500 (750–2500)

*BMI* body mass index, *PASI* psoriasis area severity index, *SD* standard deviation

**Table 2:** Laboratory data^a^ of the studied participants

Variables	Mean ± SD or Median (IQR)
HB	11.6 ± 1.34
WBC	6.5 ± 1.5
PLT	263 ± 64.3
RBG	114.7 ± 18.8
ALT	40.5 ± 14.4
AST	35 ± 11.7
Albumin	4.7 ± 3.8
Bilirubin	0.9 ± 0.1
PT	12.1 ± 0.18
Creatinine	0.9 ± 0.16
Urea	26.6 ± 5.4
Cholesterol	190.5 ± 31
TRI	134.7 ± 28
CAP in dB/m	42 (27.25–257.5)
Liver stiffness (KPa)	4.25 (2.5–8.1)

*Hb* hemoglobin, *WBC* white blood cells, *PLT* platelets, *RBG* random blood sugar, *ALT* alanine transaminase, *AST* aspartate transaminase, *PT* prothrombin time, *TRI* triglycerides, *CAP* controlled attenuation parameter in decibel/meter (dB/m), *KPa* Liver stiffness in kilopascals, *SD* standard deviation, *IQR* interquartile range

^a^Reference ranges: Hb: 12–16 g/dL; WBC: 4.0–11.0 × 10^9^/L; PLT: 150–400 × 10^9^/L; ALT: 10–40 IU/L; AST: 10–40 IU/L; RBG: 70–140 mg/dL; Total cholesterol: < 200 mg/dL; Triglycerides: < 150 mg/dL; Urea: 15–45 mg/dL; Creatinine: 0.6–1.2 mg/dL

**Table 3:** Incidence of NASH

Incidence of NASH	N (%) (*n* = 80)
NASH	16 (20)
NAFLD	21 (26.2)
Normal (non-NASH non-NAFLD)	43 (53.8)

*NASH* non-alcoholic steatohepatitis, *NAFLD* non-alcoholic fatty liver disease

Among psoriatic patient diagnosed with NASH, the mean PASI score was 8.8 ± 3.6 which was higher than those diagnosed with NAFLD and non-NASH non-NAFLD patients (6.7 ± 4.5 and 7.1 ± 3.9, respectively); however, this difference was not statistically significant (*P* = 0.19). The percentage of moderate and severe psoriasis was higher in NASH patients (68.75%) compared to NAFLD and non-NASH non-NAFLD subjects (57.1% and 46.5%, respectively), but the difference was not statistically significant (*p* = 0.27; Table 4). Although the difference in PASI scores among NASH, NAFLD, and non-NASH non-NAFLD groups did not reach statistical significance (*p* = 0.19), the observed trend toward higher PASI in NASH patients may hold clinical relevance, suggesting a possible link between skin disease severity and hepatic involvement. To provide further context, 95% confidence intervals for PASI scores were calculated and included, reflecting a directional tendency that merits validation in larger studies.

**Table 4:** Association between the NASH, NAFLD, and non-NASH non-NAFLD participants in terms of the PASI score

PASI	NASH (*N* = 16)	NAFLD (*N* = 21)	Normal (Non-NASH non-NAFLD) (*N* = 43)	*P* value
Mean ± SD	8.8 ± 3.6	6.7 ± 4.5	7.1 ± 3.9	0.19^a^
Mild	5 (31.25%)	9 (42.8%)	23 (53.4%)	0.27^b^
Moderate	8 (50%)	7 (33.3%)	10 (23.2%)	
Severe	3 (18.75%)	5 (23.8%)	10 (23.2%)	

*NASH* non-alcoholic steatohepatitis, *NAFLD* non-alcoholic fatty liver disease, *PASI* psoriasis area severity index, *SD* standard deviation

^a^One-way analysis of variance

^b^Chi-square test

*Significant at *P* < 0.05

NASH, NAFLD, and non-NASH non-NAFLD subjects were all comparable in terms of their gender and BMI (*P* = 0.6 and 0.2, respectively); however, age was significantly higher in NASH than NAFD, and non-NASH non-NAFLD subjects (48.2 ± 8.4, 45.8 ± 9.9, and 39.6 ± 10.6; *P* = 0.006). Among patients diagnosed with NASH, 56.3% had a known diagnosis of type 2 diabetes mellitus, compared to 38.1% in the NAFLD group and 20.9% in non-NASH non-NAFLD patients. BMI ≥ 30 kg/m^2^ (obesity) was observed in 62.5% of NASH patients versus 38.1% and 27.9% in NAFLD and non-NASH non-NAFLD groups, respectively (Table 5).

**Table 5:** Association between the NASH, NAFLD, and non-NASH non-NAFLD participants in terms of demographics

Variables	NASH	NAFLD	Non-NASH NAFLD	*P* value
Age (years)	48.2 ± 8.4	45.8 ± 9.9	39.6 ± 10.6	0.006*
Gender—males (%)	11 (68.8%)	12 (57.1%)	24 (55.8%)	0.6
Gender—females (%)	5 (31.1%)	9 (42.9%)	19 (44.2%)	0.6
BMI (kg/m^2^)	30 ± 3.4	28.5 ± 3.8	28.1 ± 4.3	0.2
Diabetes—*n* (%)	9 (56.3%)	8 (38.1%)	9 (20.9%)	0.01*
Obesity (BMI ≥ 30)—*n* (%)	10 (62.5%)	8 (38.1%)	12 (27.9%)	0.03*

*NASH* non-alcoholic steatohepatitis, *NAFLD* non-alcoholic fatty liver disease, *BMI* body mass index

^a^One-way analysis of variance

^b^Chi-square test

*Significant at *P* < 0.05

Random blood glucose (RBG) was significantly higher in psoriasis patients with NASH (136.1 ± 18.4 mg/dL) versus 126.4 ± 18.7 mg/dL in NAFLD and 108.8 ± 11.7 mg/dL in non-NASH non-NAFLD patients (*P* = 0.001). ALT and AST were significantly higher in NASH patients than NAFLD and non-NASH non-NAFLD patients (*P* = 0.001). Urea was significantly higher in NASH and NAFLD patients than non-NASH non-NAFLD (*P* = 0.001). On the other hand, cholesterol was significantly higher in NAFLD than NASH and non-NASH non-NAFLD patients (*P* = 0.001). The triglyceride level was significantly higher in NASH patients than others (*P* = 0.001), while the mean CAP and liver stiffness were significantly higher in NASH when compared to NAFLD and non-NASH non-NAFLD subjects (*P* = 0.001; Table 6).

**Table 6:** Association between the NASH, NAFLD, and non-NASH non-NAFLD participants in terms of laboratory data^a^

Variables	NASH (*N* = 16)	NAFLD (*N* = 21)	Non-NASH non-NAFLD (*N* = 43)	*P* value ^b^
Hb	11.8 ± 1.3	11.3 ± 1.2	11.6 ± 1.3	0.4
WBC	6.4 ± 1.5	6.8 ± 1.4	6.3 ± 1.6	0.4
PLT	260.11 ± 82.4	254 ± 46.2	286.9 ± 62.3	0.1
RBG	136.1 ± 18.4	126.4 ± 18.7	108.8 ± 11.7	0.001*
ALT	55.4 ± 12.6	49.6 ± 11.7	30.5 ± 6.3	0.001*
AST	44.8 ± 7.9	44.1 ± 13.5	27.7 ± 5.5	0.001*
Albumin	4.3 ± 0.35	4.2 ± 0.39	5.2 ± 5.2	0.5
Bilirubin	0.9 ± 0.17	0.9 ± 0.15	0.9 ± 0.12	0.3
PT	12.2 ± 0.1	12.2 ± 0.2	12.1 ± 0.14	0.6
Creatinine	1 ± 0.1	0.9 ± 0.2	0.9 ± 0.15	0.6
Urea	27.8 ± 4.8	27.5 ± 6.2	25.6 ± 5	0.002*
Cholesterol	199.2 ± 33.1	205.8 ± 24.1	179.8 ± 29.6	0.001*
TRI	156.8 ± 30	144.8 ± 28.5	121.6 ± 19	0.001*
CAP in dB/m	189.3 ± 30.3	338.9 ± 49.4	29.5 ± 9.6	0.001*
Liver stiffness in KPa	9.1 ± 0.7	7 ± 0.6	2.7 ± 0.9	0.001*

*Hb* hemoglobin, *WBC* white blood cells, *PLT* platelets, *RBG* random blood sugar, *ALT* alanine transaminase, *AST* aspartate transaminase, *PT* prothrombin time, *TRI* triglycerides, *CAP* controlled attenuation parameter in decibel/meter (dB/m), *KPa* Liver stiffness in kilopascals

*Significant at *P* < 0.05

^a^Reference ranges: Hb: 12–16 g/dL; WBC: 4.0–11.0 × 10^9^/L; PLT: 150–400 × 10^9^/L; ALT: 10–40 IU/L; AST: 10–40 IU/L; RBG: 70–140 mg/dL; Total cholesterol: < 200 mg/dL; Triglycerides: < 150 mg/dL; Urea: 15–45 mg/dL; Creatinine: 0.6–1.2 mg/dL

^b^One-way analysis of variance

**Fig. 3:**
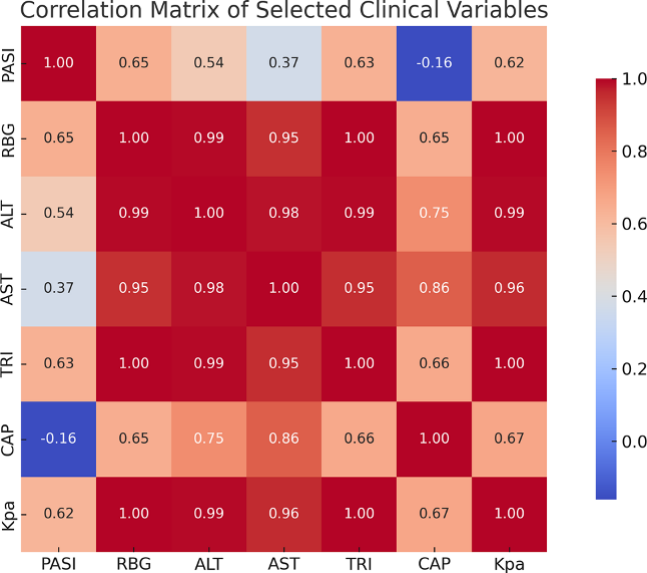
Correlation matrix heatmap illustrating relationships among clinical, metabolic, and hepatic variables in psoriasis patients. Strong positive correlations were observed between liver stiffness (KPa) and RBG, ALT, and triglycerides. PASI showed moderate correlation with both liver stiffness and RBG. Color scale reflects the strength and direction of Pearson correlation coefficients

A correlation matrix analysis was performed to assess the relationships between clinical and metabolic parameters across the study groups. Notably, liver stiffness (KPa) showed strong positive correlations with RBG (r = 0.99), ALT (r = 0.99), and triglycerides (r = 1.00), while PASI correlated moderately with KPa (r = 0.62) and RBG (r = 0.65). These findings underscore the interplay between psoriasis severity, glycemic control, and hepatic dysfunction (Fig. 3).

## Discussion

The association between psoriasis and systemic comorbidities has been well-documented, but the specific link between psoriasis and NAFLD, particularly its more severe form, NASH, remains under explored [1–3]. The current study aimed to evaluate the incidence of NASH in patients with psoriasis vulgaris and explore the relationship between psoriasis severity and liver health to provide insights into patient management and interdisciplinary care. Recent consensus proposes replacing NAFLD with metabolic associated fatty liver disease (MAFLD), emphasizing the metabolic dysfunction that underlies these conditions. Our study aligns with this paradigm, though we have retained traditional terminology for consistency with diagnostic methods used during the study period [7].

Psoriasis patients frequently exhibit metabolic abnormalities, including obesity, dyslipidemia, and insulin resistance, all of which are key risk factors for NAFLD and NASH. Additionally, systemic inflammation—a hallmark of psoriasis—plays a pivotal role in the pathogenesis of hepatic steatosis and fibrosis [8]. Previous studies have demonstrated a higher prevalence of NAFLD in psoriasis patients, but limited data exist regarding the incidence of NASH and its relationship with psoriasis severity [9, 10].

The current study identified 20% of psoriasis vulgaris patients were diagnosed with NASH, while 26.2% had NAFLD, and 53.8% had no detectable liver abnormalities. The mean PASI score, an indicator of psoriasis severity, was higher in NASH patients (8.8 ± 3.6) compared to those with NAFLD (6.7 ± 4.5) and normal liver states (7.1 ± 3.9), although the difference did not reach statistical significance (*P* = 0.19). Furthermore, moderate-to-severe psoriasis was more prevalent among NASH patients (68.75%) than in NAFLD (57.1%) and non-NASH non-NAFLD patients (46.5%). These observations suggest a potential association between psoriasis severity and the progression to NASH, consistent with prior research linking systemic inflammation to hepatic dysfunction [6, 8].

Our study revealed a significant difference in age between the three groups, with NASH patients being older (48.2 ± 8.4 years) than those with NAFLD (45.8 ± 9.9 years) and non-NASH non-NAFLD subjects (39.6 ± 10.6 years, *P* = 0.006). This aligns with existing literature emphasizing age as a risk factor for both psoriasis-related systemic complications and NASH development [11, 12]. However, BMI and gender were not significantly different between the groups (*P* = 0.2 and *P* = 0.6, respectively), suggesting that factors beyond traditional metabolic risk markers may contribute to the observed hepatic outcomes.

Biochemical analyses highlighted key distinctions between the groups. Random blood glucose (RBG) levels were significantly elevated in NASH patients (136.1 ± 18.4 mg/dL) compared to NAFLD (126.4 ± 18.7 mg/dL) and normal patients (108.8 ± 11.7 mg/dL, *P* = 0.001). Similarly, liver enzymes (ALT and AST) were markedly higher in NASH patients (*P* = 0.001), indicating more pronounced hepatic inflammation. Elevated triglycerides (TRI) and urea levels in NASH patients further underscore the metabolic dysfunction underlying this condition (*P* = 0.001 for both). These findings are consistent with earlier studies identifying metabolic syndrome and insulin resistance as critical contributors to NASH pathogenesis in psoriasis patients [13, 14].

Conversely, cholesterol levels were significantly higher in NAFLD patients compared to NASH and non-NASH non-NAFLD groups (*P* = 0.001). This discrepancy may reflect differing metabolic pathways or degrees of lipid dysregulation between simple steatosis and NASH. Furthermore, CAP and KPa values, indicative of hepatic steatosis and stiffness, respectively, were significantly elevated in NAFLD patients, suggesting that simple steatosis remains a predominant feature in this subgroup (*P* = 0.001 for both). The present findings, which link higher PASI scores to increased NASH incidence, align with prior studies emphasizing the role of systemic inflammation in bridging psoriasis severity and liver disease [15]. Additionally, the higher prevalence of moderate-to-severe psoriasis in NASH patients supports the hypothesis that greater inflammatory burden may accelerate hepatic disease progression [16, 17].

The findings highlight a significant burden of NASH and NAFLD among psoriasis patients, underscoring the shared pathogenic pathways linking these conditions. The elevated incidence of NASH in this cohort likely reflects the cumulative effects of systemic inflammation, metabolic dysregulation, and hepatic steatosis, which are central to both psoriasis and NAFLD pathogenesis [18].

Several cytokines implicated in psoriasis pathogenesis, particularly interleukin-17 (IL-17) and tumor necrosis factor-alpha (TNF-α), are also known to contribute to hepatic inflammation and fibrogenesis [18, 19]. These proinflammatory mediators can promote insulin resistance, lipotoxicity, and stellate cell activation, thereby linking cutaneous and hepatic pathology through systemic inflammatory pathways [19]. These cytokines promote insulin resistance and disrupt lipid metabolism, contributing to the progression from simple steatosis to NASH [19]. The higher mean PASI scores in NASH patients suggest that greater psoriasis severity may correlate with increased hepatic inflammation and injury. However, the lack of statistical significance in PASI score differences may indicate that additional factors, such as genetic predisposition or environmental influences, modulate the psoriasis-NASH relationship [19]. Several shared mechanisms may underlie the psoriasis–NASH connection. Inflammatory cytokines such as IL-17, IL-23, and TNF‑α not only sustain cutaneous lesions but also promote hepatic insulin resistance and fibrogenesis. Genetic susceptibility loci such as PNPLA3 variants and IL‑6 pathway polymorphisms have been linked to both diseases, suggesting a shared pathophysiological basis [17–19].

Age emerged as a significant factor, with older patients exhibiting higher rates of NASH. This finding aligns with the natural history of NAFLD, where age-related metabolic changes and prolonged inflammatory exposure contribute to hepatic fibrosis [20]. Elevated RBG and liver enzymes in NASH patients further support the role of metabolic dysfunction in disease progression, while the absence of significant differences in BMI suggests that psoriasis-specific mechanisms may independently influence NASH development [21].

Our findings are consistent with previous studies demonstrating a high prevalence of NAFLD and NASH in psoriasis patients. Roberts et al. [10] determined the prevalence of both NAFLD and non-alcoholic steatohepatitis (NASH) in patients with psoriasis. A total of 129 patients were enrolled and 103 completed all necessary studies. They found that the overall prevalence of NAFLD was 47% and the overall prevalence of NASH was 22% in those who underwent biopsy. Gisondi et al. [8] reported a strong association between psoriasis and NAFLD, particularly in patients with severe disease and similarly, Miele et al. [22] highlighted the role of systemic inflammation in linking psoriasis and hepatic steatosis.

However, the lack of statistical significance in PASI score contrasts with some studies that suggest a linear relationship between psoriasis severity and NAFLD/NASH risk [11, 23]. This discrepancy may stem from differences in study design, sample size, or patient characteristics. Additionally, the higher cholesterol levels observed in NAFLD patients compared to NASH and normal groups deviate from typical lipid profiles reported in NASH studies, highlighting the need for further investigation into lipid metabolism nuances in psoriasis patients [24].

This study’s strengths include its detailed assessment of liver health using both imaging and histological criteria, providing robust data on NASH incidence in psoriasis patients. Additionally, the inclusion of biochemical and metabolic parameters enables a comprehensive evaluation of contributing factors. The presence of comorbid conditions such as diabetes mellitus, hypertension, and dyslipidemia—each of which was documented in our cohort—may act as potential confounders influencing liver pathology. While our subgroup analysis identified trends in these variables, the study was not powered to fully adjust for their effects. Future studies employing multivariate models are needed to clarify the independent role of psoriasis severity in NASH development.

However, several limitations warrant consideration. The cross-sectional design precludes establishing causal relationships between psoriasis severity and NASH. Moreover, the absence of biopsy-verified fibrosis in our cohort remains a limitation. The relatively small sample size may limit the generalizability of findings, and the lack of longitudinal follow-up prevents assessment of disease progression over time.

Additionally, inflammatory biomarkers such as CRP (C reactive protein), ESR (erythrocyte sedimentation rate), IL‑6, or TNF‑α were not measured, which limits our ability to directly assess systemic inflammation. Future studies should include these to further explore inflammation-driven pathways between psoriasis and liver disease. Due to the modest sample size, we were unable to perform a multivariate logistic regression to adjust for potential confounders such as age, BMI, and glycemic or lipid parameters. Future studies with larger cohorts are needed to confirm whether psoriasis severity independently predicts NASH risk. As liver biopsy was not performed, we acknowledge the inability to definitively distinguish NASH from drug-induced liver fibrosis using ultrasonography and transient elastography alone.

## Conclusion

A burden of NASH and NAFLD in patients with psoriasis vulgaris was identified, with potential links to psoriasis severity and systemic metabolic dysfunction. These findings underscore the importance of interdisciplinary care and proactive liver screening in managing this high-risk population. While patients with NASH had higher PASI scores than those in other groups, this trend did not reach statistical significance and should be interpreted with caution. Clinicians should consider liver screening in psoriasis patients with moderate-to-severe disease or those with metabolic risk factors using non-invasive tools such as ultrasonography or FibroScan. Future studies with larger cohorts and prospective designs are needed to validate these findings and explore underlying mechanisms. As this was a cross-sectional study, no causal relationships can be inferred. Longitudinal studies are needed to explore temporal links between psoriasis severity and liver disease progression.

## Data Availability

All authors confirm all data and materials as well as software applications or custom code support their published claims and comply with field material.

## Abbreviations

CAP: Controlled attenuation parameter
IL-17: Interleukin 17
KPa: Kilopascal
LSM: Liver stiffness measurement
NAFLD: Non-alcoholic fatty liver disease
NASH: Non-alcoholic steatohepatitis
PASI: Psoriasis Area Severity Index
RBG: Random blood glucose
TNF‑α: Tumor necrosis factor-alpha

## Availability of data

Data that support the findings of this study are available from the corresponding author upon reasonable request.
